# Prevalence of *Salmonella* by Serological and Direct Detection Methods in Piglets from Inconspicuous, Conspicuous, and Vaccinated Sow Herds

**DOI:** 10.3390/ani10010029

**Published:** 2019-12-21

**Authors:** Juhle-Marijke Buch, Christian Visscher, Anton Schulte zu Sundern, Josef Schulte-Wülwer, Ansgar Deermann, Carolin Holling

**Affiliations:** 1 Institute for Animal Nutrition, University of Veterinary Medicine Hannover, Foundation, Bischofsholer Damm 15, D-30173 Hanover, Germany; juhle-marijke.buch@tiho-hannover.de (J.-M.B.); anton.schulte.zu.sundern@tiho-hannover.de (A.S.z.S.); 2 EVH Select GmbH, An der Feuerwache 14, D-49716 Meppen, Germany; j.schulte-wuelwer@kabelmail.de (J.S.-W.); deermann@porcussanus.de (A.D.); 3 Schweinegesundheitsdienst der Landwirtschaftskammer Niedersachsen, Fachbereich Tiergesundheit, Hermann-Ehler-Str. 15, D-26160 Bad-Zwischenhahn-Wehnen, Germany; carolin.holling@lwk-niedersachsen.de

**Keywords:** salmonella, pig farms, vaccination, prevalence, serology

## Abstract

**Simple Summary:**

*Salmonella* is one of the most important bacterial zoonotic pathogens worldwide that can lead to infections in humans, particularly through the consumption of contaminated food. The percentage of diarrhoeal diseases attributable to the consumption of pork products has risen in recent years, whereas cases associated with poultry meat have considerably decreased. In the present study, the *Salmonella* prevalence on piglet-producing farms was determined on the basis of environmental samples and blood samples on 24 pig farms previously classified as *Salmonella*-inconspicuous (SI) and *Salmonella*-conspicuous (SC). In addition, the effects of sow vaccination against *Salmonella* prevalence in piglets on SC farms were investigated. The evaluation confirmed the previous classification into SC and SI farms—SC-farms showed significantly higher *Salmonella* prevalence in environmental samples and significantly higher OD% values in blood samples from piglets. Furthermore, vaccination of sows on SC farms was accompanied by the highest *Salmonella* prevalence in the environment, and by the highest antibody titer values in piglets, and therefore cannot influence the Salmonella prevalence solely at the farm level.

**Abstract:**

Due to the zoonotic potential of *Salmonella*, the high prevalence of *Salmonella* on pig farms deserves particular attention. Because there is limited precise data on piglet-producing farms, this survey evaluated the *Salmonella* status of 24 different pig farms that had previously been divided into 12 *Salmonella*-conspicuous (SC) and 12 *Salmonella*-inconspicuous (SI) farms on the basis of the serological status of their piglets (25 kg). The evaluation was based on 498 environmental samples and 2641 blood samples, as well as on a biosecurity screening. SC farms were subdivided into farms with sow vaccination against *Salmonella* (*n* = 3) and those without vaccination (*n* = 9). In accordance with the previous classification, both the highest *Salmonella* prevalence in the environment and the highest antibody titers of the examined piglets were determined on SC farms at both defined time points. Piglets from vaccinated sows showed the highest OD% values, before and after vaccination. On SC farms, most *Salmonella*-positive samples could be obtained in rearing areas (2017: 40.8%, 2019: 26.0%). The results of this study indicate that sow vaccination alone cannot influence *Salmonella* prevalence at the farm level. Above all, general infection pressure seems to play a major role for *Salmonella* prevalence in the environment and for high OD% values of related pigs.

## 1. Introduction

*Salmonella* is one of the most important bacterial zoonotic and coincident food-borne pathogens worldwide and therefore one of the main causes of food-borne diseases in humans, especially caused by the serovars *Salmonella enteritidis* and *Salmonella typhimurium* [1,2]. At European the level, no significant increase or decrease in the number of human salmonellosis cases has been observed over the last years (2013–2017) [2]. In 2017, the declining trend of human cases of salmonellosis in Germany (14,269 cases) did not continue for the first time in recent years [3]. However, the slightly lower number of cases in 2018 (13,529 cases) still remained above the reported number of infections in 2016 [1]. Nevertheless, these published figures presumably do not reflect the actual number of cases, as it must be assumed that a large number of diagnosed cases of salmonellosis are not further investigated and documented with regard to their origin and, despite the high probability of a food-associated infection occurring, these do not appear as such in the case numbers [3]. Given that most *Salmonella* infections in pigs are subclinical and prevalent among all age groups and in different production stages, identifying infected pigs on farms can be difficult and costly [4]. Due to the demonstrated high correlations between *Salmonella* prevalence on pig farms and carcass contamination, control mechanisms at the farm level are of major importance [5,6]. At the level of fattening farms, a legally prescribed monitoring programme [7], which has its private counterpart in quality assurance monitoring (“QS-Salmonellenmonitoring”), provides comprehensive data on the situation of farms at the end of the “pre harvest section”. However, as some studies have already identified the acquisition of asymptomatic carriers as the main source of *Salmonella* in herds [8,9,10], and no related monitoring programmes have involved the piglet producers so far, there is a need to investigate appropriate approaches to reducing cases of *Salmonella* already in piglet rearing at the beginning of the chain [11,12]. Therefore, epidemiological and bacteriological investigations for *Salmonella* are of great importance in order to identify a general *Salmonella* status on piglet-producing farms. For control mechanisms, vaccines are described as the primary tool for disease prevention in veterinary animal species, with these being essential for helping maintain animal welfare and productivity [13]. Vaccines can be used to control clinical diseases whereby in modern pig production, vaccination is mainly used to reduce infection pressure by suppressing the shedding of *Salmonella* [14]. Because a complete prevention of *Salmonella* colonisation in pigs by vaccination would require freedom from *Salmonella* in early age accommodation and among dams, vaccination can only be a supportive measure [14]. In the present study, *Salmonella* prevalence on piglet-producing farms was investigated using blood samples and environmental samples with regard to distribution on the farm, compliance with previous classification into *Salmonella*-inconspicuous and *Salmonella*-conspicuous farms, as well as effects of sow immunisation on *Salmonella* prevalence in environmental samples and on optical density (OD%) values of the piglets.

## 2. Materials and Methods 

### 2.1. Design of the Study

**Figure 1:**
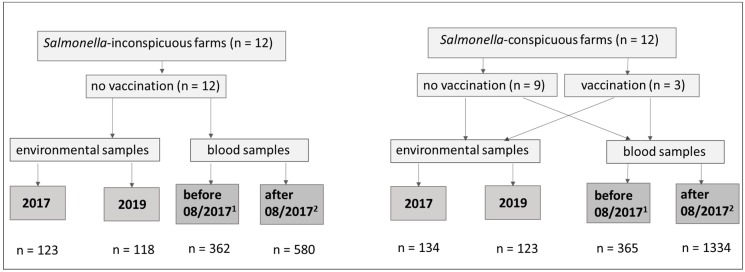
Trial flow diagram. The diagram indicates the distribution of selected farms, the sample scheme, and the number of taken samples, ^1^ January 2016 to July 2017, ^2^ August 2017 to July 2019.

The present study was carried out from 2016 to 2019 on 24 (f1–f24) pig farms in Lower Saxony (Germany) in cooperation with the EVH-Select GmbH (association of six piglet producer communities in Lower Saxony; Figure 1). The farms in the present study had previously been classified into 12 *Salmonella*-inconspicuous (SI) and 12 *Salmonella*-conspicuous (SC) farms on the basis of retrospective evaluation of data from a voluntary health screening organised by the EVH-Select GmbH on approximately 230 farms [15]. Only farms that had regularly participated in the health screening over the previous 3 years (at least three times) were selected. For the health-screening [16], 10 piglets (~25 kg, end of rearing period, ~70 d.) were used on each farm. The piglets used for sampling were randomly selected. Blood samples of each piglet were examined for *Salmonella* lipopolysaccharide (LPS) antibodies, detected by Herdcheck *Salmonella* ELISA (IDEXX Laboratory, Hoofddorp, the Netherlands). With an optical density (OD) ≥ 10% the samples were considered “positive”. Farms that had experienced the highest *Salmonella* seroprevalence of ready-to-sell-piglets for a longer period were classified as SC (*n* = 12). An additional 12 SI farms were selected, comparable to the SC farms in hygiene, management, performance, farm size, and veterinary care. The sows of three SC farms were vaccinated in August 2017 against *S. typhimurium* with the live attenuated vaccine SALMOPORC (IDT Biologica GmbH, Dessau-Roßlau, Germany) after the first round of sampling. For the evaluation, 12 SC and 12 SI farms were examined with regard to their *Salmonella* status by taking environmental samples and individual blood samples.

### 2.2. Animals 

The participating farms were farrow-to-feeder-farms for the most part (*n* = 22) with a small proportion of farrow-to-finisher-farms (*n* = 2). The breeding lines of the sows varied from lines of DanAvl, Herlev, Dänemark (*n* = 10), lines of the Bundes Hybrid Zucht Programme, Ellringen, Germany (BHZP, *n* = 7); Topig’s Norsvin, Senden, Germany (*n* = 3); or Pig Improvement Company Deutschland GmbH, Hannover, Germany (PIC, *n* = 4). For boar lines PIC 408, Hannover, Germany (*n* = 8), db.77 (*n* = 6), German Pietrain, Stuttgart-Plieningen, Germany (*n* = 3), Topig’s (*n* = 3), and others (n = 4) were used. Most of the farms produced at three-weekly intervals (*n* = 9), followed by four-weekly intervals (*n* = 6), weekly-intervals (*n* = 4), and other intervals (*n* = 5). The number of sows per farm varied from 105 sows to 724 sows with an average of 310 sows, and the number of boars per farm varied from 1 boar to 4 boars with an average of 2 boars. Sows of farm f3, f4, and f11 were vaccinated with the commercial *S. typhimurium* live vaccine “SALMOPORC” (IDT Biologica GmbH, Dessau-Roßlau, Germany) in August 2017 (initial immunization: w 6 and 3 ante partum (a. p.), afterwards w 3 a. p.). The time of sow vaccination thus divided the sampling time into “before” and “after” vaccination.

### 2.3. Sample Collection

A total of 498 environmental samples were collected on the 24 farms at different time points in 2017 and 2019 from different areas intra-farm, whereby care was taken to obtain samples from areas of all age groups in addition to samples from storage and areas between. On average, 10.4 ± 1.1 samples per farm and points in time were taken, distributed over vestibules/corridors/offices, sow-areas, farrowing-areas, rearing-areas, and gilt areas, as well a as few samples in the farrowing and rearing area after cleaning and disinfection. On the three farms with sow vaccination (f3, f4, f11), sampling in 2017 was carried out prior to the first time of vaccination. During sampling in 2019, all sows on these three farms were vaccinated.

For serological examination, blood samples from 10–15 randomly selected piglets (28–30 kg) on each farm (f1–f24) were tested for antibodies at each interval (three-weekly, four-weekly, etc.) from the beginning of 2016 to mid-2019 continuously (2641 samples in total). Blood samples with an optical density of ≥10% were considered “positive”. The sampling period was divided into “before 08/2017” and “after 08/2017”, since in August 2017 the sows on farm 3, 4, and 11 were vaccinated. 

### 2.4. Tests for Salmonella

Environmental samples were culturally analysed by the Institute for Microbiology, University of Veterinary Medicine Hannover, Foundation, Hannover, Germany, and by Vaxxinova diagnostics GmbH, Leipzig, Germany. The samples were obtained using sock and environmental swabs that were pre-enriched with Buffered Peptone Water (BPW) for 18 h (37 °C) with a following selective enrichment step in Rappaport Vassiliadis selective enrichment medium (RV, Oxoid, Basingstoke, Hampshire, UK) and tetrathionate brilliant green bile broth (TBG, Merck, Darmstadt, Germany) for 24 h and 48 h, respectively. Suspicious colonies were confirmed by sub-cultivation on selective culture medium Brilliance-Salmonella (Thermo Scientific, Wesel, Germany). All blood samples were examined by Vaxxinova diagnostics GmbH, Leipzig, Germany, using the Herdcheck *Salmonella* ELISA (IDEXX Laboratory, Hoofddorp, The Netherlands) for detecting *Salmonella* LPS antibodies.

### 2.5. Biosecurity 

To gain an impression of the hygiene and management standards on farm, a biosecurity screening was carried out from November 2016 until February 2017 on every holding, following a standard biosecurity protocol (www.biocheck.ugent.be). In this screening, external and internal biosecurity aspects were analysed and evaluated [17]. External biosecurity focuses on every aspect that involves contact with the outside world and can be divided into six subunits: purchase of piglets, transport of animals and removal of manure and carcasses, feed and water and material supply, entrance of visitors and personnel, vermin and bird control, and location and environment. Internal biosecurity focuses on all aspects on the farm, being subdivided into disease management, farrowing and suckling period, nursery unit, fattening unit, compartmentalising and working lines and equipment, and cleaning and disinfection. All parameters were collected by means of a questionnaire covering all subgroups of external and internal biosecurity aspects. The answers were then used to make a percentage assessment of biosecurity at the farm level. 

### 2.6. Statistical Analysis

Data were statistically analysed using the SAS Enterprise Guide (version 7.1, Fa. SAS Institute Inc., Cary, NC, USA). Differences regarding the number of *Salmonella*-positive environmental samples and the number of blood samples with an optical density of ≥10% were analysed using the chi-square homogeneity test. For evaluating the biosecurity levels, a one-way ANOVA test was used. The correlation between *Salmonella* prevalence in the environment and the OD% values of the piglets were calculated using Sperman’s correlation coefficient.

## 3. Results

### 3.1. Salmonella Prevalence in the Environment

The evaluation was based on 498 environmental samples, taken on 24 different piglet-producing farms in Lower Saxony. The number of *Salmonella*-positive samples found on farms previously classified as SC was significantly higher (2017: 20.1%, 2019: 14.6%) than on farms previously classified as SI (2017: 4.88%, 2019: 3.39%). On SC farms, the highest prevalence of *Salmonella* was observed in the rearing area with 40.8% *Salmonella*-positive samples in 2017 and 26.0% in 2019, followed by “areas between” (vestibules/corridors/offices) with 20.0% in 2017 and 16.7% in 2019. On SI farms, no *Salmonella*-positive samples were found in farrowing areas (Table 1).

With the exception of one farm (f3), there were no significant differences in number of *Salmonella*-positive samples between 2017 and 2019, although on most farms a numerical decrease in positive samples could be observed (Table 2).

### 3.2. Optical Densities (OD%) of the Examined Blood Samples

According to the number of positive blood samples from piglets weighing 25 kg, significant differences were observed. Although the significantly highest number of blood samples with an optical density ≥ 10% could be observed in piglets from vaccinated sows on farms 3, 4, and 11 (SC-farms), the lowest number could be observed in piglets from SI farms (Table 3). All blood samples of piglets from SC farms combined (farms with and without sow vaccination) had an average optical density of 5.77% before August 2017 and 10.12% after August 2017, and all blood samples of piglets from SI-farms had an average of 6.73% OD before August 2017 and 2.47% OD after August 2017. On SI and SC farms, a significant decrease in positive blood samples between 2017 and 2019 was observed, whereas on vaccination farms, a significant increase in positive blood samples was detected.

### 3.3. Biosecurity Check

**Figure 2:**
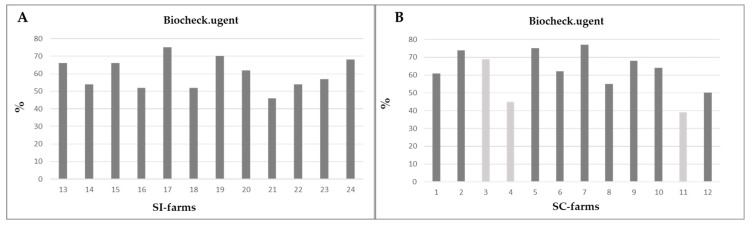
Comprehensive biosecurity evaluation of all 24 farms, data shown as a percentage. (**A**) SI farms; (**B**) SC farms; 3, 4, 11 = farms with sow vaccination.

The evaluation of the biosecurity screening showed significant differences (*p* < 0.05) in the internal biosecurity parameters—although SI farms had an average internal biosecurity of 59.17 ± 16.80%, SC farms had an average of 70.92 ± 6.91%. There were no significant differences in the external and comprehensive biosecurity between SI and SC farms (SI farms: external biosecurity = 56.00 ± 18.73%, comprehensive biosecurity = 60.17 ± 8.89%; SC farms: external biosecurity = 53.25 ± 16.78%, comprehensive biosecurity = 61.58 ± 12.20%; Figure 2).

## 4. Discussion

At the European Level (EU), Regulation (EC) No. 2160/2003 sets community targets for reducing the prevalence of *Salmonella* and other zoonotic agents that may be transmitted through contaminated food [18]. With the introduction of the Pig-Salmonella-Regulation in Germany [7], the mandatory participation in the *Salmonella* monitoring programme of pig-fattening farms with more than 50 fattening places is required. Piglet producers, however, are not involved in a comprehensive monitoring programme, which is why there is limited precise data available on *Salmonella* prevalence on farrow-to-feeder farms so far [12,15]. In order to obtain a more accurate picture, epidemiological and bacteriological investigations for *Salmonella* are of great importance.

The retrospective analysis of the *Salmonella* status of the 24 concerned farms was based solely on OD% value evaluation of blood samples. No direct *Salmonella* detection had been carried out, and therefore no statement concerning the environmental *Salmonella* prevalence was available [15]. First, the optical density values tested in blood samples of piglets (28–30 kg) on each farm confirmed the previous classification. Piglets from SC farms showed significantly higher OD% values than those from SI farms, even though antibody titers remained at a relatively low level in general. The additionally examined 498 environmental samples from all 24 farms were in line with these results—on farms previously classified as SC, significantly more *Salmonella*-positive samples could be obtained (2017: 20.1%; 2019: 14.6%) compared to SI farms (2017: 4.88%; 2019: 3.39%). A numerically recognisable reduction in positive samples in 2019 could not be statistically confirmed. An investigation into *Salmonella* prevalence on U.K. pig farms showed similar interrelations—in a *Salmonella* surveillance programme, pig farms had been classified as “platinum” when they maintained a low seroprevalence (<10%) [19]. On three farms that had lost their platinum status, environmental samples and pooled faecal swaps were taken over a period of 15 months where, similar to our results, all farms remained positive with an overall prevalence between 19% and 46% [19]. A pan-European study in 24 member states and two non-member states showed a total *Salmonella* prevalence of 31.8% on holdings with breeding pigs in 2008, whereby no previous distinction between conspicuous and inconspicuous farms had been made [20]. Schulte zu Sundern on the other hand investigated OD% values of corresponding sows on farms from the study discussed here [15]—contrary to the previous classification, the percentage of serological positive samples (OD > 40%) on SI farms was on average 40.9% (Ø 45.43 OD%), and 29.6% (Ø 32.88 OD%) on SC farms. Hence, the question was raised as to whether the exclusive examination of piglets in the origin analysis for health status could provide a realistic picture of *Salmonella* prevalence in the entire herd [15]. Our results merely give an insight into the situation in rearing areas of piglets up to 30 kg. On SC farms especially, rearing areas showed a high prevalence of *Salmonella* in the environment, compliant with higher OD% values of the concerned piglets in these areas compared to those on SI farms. The generally higher *Salmonella* prevalence in the environment correlated with a higher OD% value in blood samples of the piglets on SC farms (correlation coefficient = 0.66434, *p* = 0.0185). On SI-farms, no similar correlation could be observed (correlation coefficient = 0.36650, *p* = 0.2413). Because the previous classification of the farms was disapproved in relation to the antibody titers of sows [15], the classification that was made appears to apply only or at least to the rearing areas. Still, irrespective of the farm classification, a high percentage of *Salmonella* antibodies could be detected in the study by Schulte zu Sundern [15] that, contrary to the situation in the rearing area, seemed not to correlate with a high *Salmonella* prevalence in the farrowing area. 

The biosecurity evaluation showed significantly higher internal biosecurity levels on SC farms compared to SI farms. As the classification of the farms took place before the biosecurity screening, individual measures were already established at the farm level when the screening started. SC farms in particular seem to have improved their hygiene protocol. Similarly, Gotter et al. showed at a first glance illogical relationships. They conducted a case–control study to determine key contributing risk factors for pig farms and could identify parameters such as having not clean boots available on the farm as being protective, whereas wearing protective clothing outside the barn increased the *Salmonella* risk [21]. The evaluation of environmental samples in the present study showed a significantly higher prevalence of *Salmonella* in the environment of SC farms, especially in rearing areas despite increased biosecurity measures. This fact clearly states the still necessary adaptation of various hygiene measures to achieve a reduction of *Salmonella* in the environment, even if certain biosecurity parameters have already been adapted. Interrelations with parameters that have not been investigated can both increase and decrease the determined risk [21]. Given that clean boots and protective clothing appear to be part of the biosecurity parameters that should ensure good hygiene [21], these already aforementioned observations underline that, despite partly improved biosecurity within the farms in our study, the reduction of *Salmonella* depends on many different hygiene aspects and further parameters.

The significantly higher number of blood samples with an optical density of ≥10% in piglets from vaccinated sows were associated with high *Salmonella* prevalence in the respective rearing areas. The aim of immunising sows is to reduce the excretion of *Salmonella typhimurium* wild strains, especially during the suckling period [22]. However, maternal antibodies in piglets only have a short half-life period [23]. In the literature, different data on the development of antibody titers can be found—Hur et al. observed a significant increase in immunoglobulin (Ig)G and IgA levels in piglets after immunisation of pregnant sows [24]. In contrast, a study on the immunisation of sows with a homologous inactivated *Salmonella* vaccine was shown to significantly decrease antibody activities of immunoglobulin (Ig)A and IgG in their offspring and, on top of this, no *Salmonella* excretion by weaners up to the age of 20 weeks was observed [25]. This would indicate a protective effect of sow vaccination on *Salmonella* excretion by their offspring due to maternal seroconversion after vaccination, similar to effects after naturally acquired infections [26]. Still, there is no certainty as to whether maternal antibodies can prevent infection and the associated formation of antibodies in piglets [23]. In different studies, the persistence of maternal antibodies is specified with approximately 8 weeks [27,28]. Thus, maternal antibodies decline before the end of the rearing period. All blood samples examined in our study came from piglets at the end of the rearing phase at approximately 70 days of age, which meant that according to the stated persistence of maternal antibodies, there was only a small likelihood of remaining maternal antibodies. Due to the overall higher *Salmonella* prevalence on SC farms, especially in rearing areas, a high probability of contact is given in this phase. As the piglets remained at the rearing area for about 6 to 8 weeks, a potential serological reaction can be expected [29]. Therefore, the significantly higher number of positive blood samples of piglets from farms with sow vaccination cannot solely be explained by remaining maternal antibodies, but rather by the higher prevalence of *Salmonella* in the environment and the correspondingly higher infection pressure, or a combination of both. Additionally it cannot be foreclosed that the increased prevalence dates from before vaccination, given that the average biosecurity value on vaccination farms was only 51%. Generally rather poor hygiene must always be included in the analysis of possible causes for a high *Salmonella* prevalence [5,30]. Because samples from only three farms with immunised dams were included in the evaluation, possible further circumstances on the respective farms have to be considered in the evaluation as well. Serological confirmation of serotypes was very limited. Only one confirmed *S. typhimurium* isolate could be identified on each of the three vaccination farms (f3: 1/6, f4: 1/11, f11: 1/5). 

## 5. Conclusions

The obtained results show the necessity of a stringent prevention of the transmission of *Salmonella* in the rearing area. Even though the evaluation of internal biosecurity measures showed significantly higher biosecurity efforts on SC farms, already implied measures do not last to be enough to reduce the already long-standing higher *Salmonella* prevalence on these farms. The collected samples of the environment as well as the blood samples from piglets on SC farms showed a significantly higher *Salmonella* prevalence compared to the investigated SI farms. If all appropriate measures were complied, the vaccination of piglets would then have to be checked in addition to the vaccination of sows in order to make a final statement regarding the possible supportive effects of vaccination on reducing the *Salmonella* prevalence. With the predominant problems and high prevalence of *Salmonella*, especially in the rearing area, no improvement of *Salmonella* status could be achieved in our investigations by the sole vaccination of sows in combination with slightly increased biosecurity measures.

Because all SC farms remained with their “*Salmonella*-conspicuous” classification, this investigation underlines the continuing need to evaluate and control the *Salmonella* prevalence already at the level of piglet production.

## Figures and Tables

**Table 1:** Number of *Salmonella*-positive environmental samples, divided into samples from *Salmonella*-inconspicuous (SI) and *Salmonella*-conspicuous (SC) farms, as well as into six different areas per farm.

	2017		2019	
	SI	SC	SI	SC
Samples (*n*)	123	134	118	123
*Salmonella*-positive (*n*)	6	27	4	18
*Salmonella*-positive (%)	4.88	20.1	3.39	14.6
Area ^1^	*Number of Positive Samples/Percentage of Positve Samples*			
1	1/8.33	4/20.0	0/0.00	1/16.7
2	2/6.06	1/3.70	1/2.85	2/5.55
3	0/0.00	1/3.33	0/0.00	2/7.69
4	2/4.44	20/40.8	3/6.52	13/26.0
5	0/0.00	1/16.7	0/0.00	0/0.00
6	1/33.3	0/0.00	0/0.00	0/0.00
Chi-square ^a^	0.0003		0.0024	

^1^ 1 = vestibules/corridors/offices, 2 = sow area, 3 = farrowing area, 4 = rearing area, 5 = gilt area, 6 = after cleaning and disinfection in farrowing and rearing areas. ^a^ Differences in the number of *Salmonella*-positive samples between SC and SI farms (both in 2017 and 2019) were analysed using the chi-square homogeneity test.

**Table 2:** Number of *Salmonella*-positive environmental samples from the examined farms.

Farm ^1^	2017		2019	
	n_pos_/n_all_	%	n_pos_/n_all_	%
1	4/12	33.0	1/10	10.0
2	0/15	0.00	0/10	0.00
3	1/11	9.10 ^b^	5/10	50.0 ^a^
4	6/12	50.0	4/11	36.4
5	3/11	27.3	0/11	0.00
6	1/10	10.0	1/10	10.0
7	3/11	27.3	5/10	50.0
8	1/9	11.1	0/9	0.00
9	1/10	10.0	0/10	0.00
10	2/11	18.2	2/10	20.0
11	2/12	16.7	0/12	0.00
12	3/10	30.0	0/10	0.00
13	0/11	0.00	1/10	10.0
14	0/10	0.00	0/10	0.00
15	0/10	0.00	0/10	0.00
16	1/10	10.0	0/10	0.00
17	0/10	0.00	2/10	20.0
18	0/11	0.00	0/10	0.00
19	2/12	16.7	1/10	10.0
20	1/11	9.10	0/10	0.00
21	0/10	0.00	0/10	0.00
22	0/10	0.00	0/10	0.00
23	0/8	0.00	0/9	0.00
24	2/10	20.0	0/9	0.00

^1^ f1–f12 = SC farms, f13–f24 = SI farms, f3, f4, f11 = farms with sow immunisation in 2017; ^a, b^ number of *Salmonella*-positive samples differ significantly within a line (*p* < 0.05), statistical differences were analysed using the chi-square homogeneity test.

**Table 3:** Number of blood samples with an optical density ≥ 10%, divided into piglets from SI farms and SC farms with and without sow vaccination.

Time	SI (*n* = 12 Farms)	SC (*n* = 9 Farms)	SC/Vaccination ^1^ (*n* = 3 Farms)
	*Number of Samples with ≥10% OD/Percentage of Samples ≥10% OD*		
Samples before 08.2017 ^2^	50/13.81 ^c, A^	107/35.67 ^b, A^	28/43.08 ^a, B^
Samples after 08.2017 ^3^	33/5.69 ^c, B^	108/27.00 ^b, B^	563/60.28 ^a, A^

^1^ Sows on farms 3, 4, and 11 were first vaccinated in August 2017; ^a, b, c^ number of samples with ≥10% optical density (OD) differ significantly within a line (*p* < 0.0001); ^A, B^ median optical densities differ significantly within a column (*p* < 0.05); statistical differences were analysed using the chi-square homogeneity test; inconspicuous = 12 farms, conspicuous = 9 farms, conspicuous + vaccination = 3 farms. ^2^ January 2016 to July 2017, ^3^ August 2017 to July 2019.
